# RiceATM: a platform for identifying the association between rice agronomic traits and miRNA expression

**DOI:** 10.1093/database/baw151

**Published:** 2016-12-26

**Authors:** Wei-Ting Liu, Chia-Chun Yang, Rong-Kuen Chen, Woei-Shyuan Jwo, Chih-Wen Wu, Wen-Yen Ting, Dah-Pyng Shung, Chun-Chi Liu, Jeremy J.W. Chen

**Affiliations:** 1 Institute of Biomedical Sciences; 2 Institute of Molecular Biology, National Chung Hsing University, Taichung, Taiwan; 3 Chiayi Branch Station, Tainan District Agricultural Research and Extension Station, Council of Agriculture, Chiayi, Taiwan; 4 Taiwan Agricultural Research Institute, Taichung, Taiwan; 5 Kaohsiung District Agricultural Research and Extension Station, Council of Agriculture, Kaohsiung, Taiwan; 6 Taitung District Agricultural Research and Extension Station, Council of Agriculture, Taitung, Taiwan; 7 Hualien District Agricultural Research and Extention Station, Council of Agriculture, Hualien, Taiwan; 8 Institute of Genomics and Bioinformatics, National Chung Hsing University, Taichung, Taiwan; 9 Agricultural Biotechnology Center, National Chung Hsing University, Taichung, Taiwan and; 10 Rong Hsing Research Center for Translational Medicine, National Chung Hsing University, Taichung, Taiwan

## Abstract

MicroRNAs (miRNAs) are known to play critical roles in plant development and stress-response regulation, and they frequently display multi-targeting characteristics. The control of defined rice phenotypes occurs through multiple genes; however, evidence demonstrating the relationship between agronomic traits and miRNA expression profiles is lacking. In this study, we investigated eight yield-related traits in 187 local rice cultivars and profiled the expression levels of 193 miRNAs in these cultivars using microarray analyses. By integrating the miRBase database, the rice annotation project database, and the miRanda and psRNATarget web servers, we constructed a database (RiceATM) that can be employed to investigate the association between rice agronomic traits and miRNA expression. The functions of this platform include phenotype selection, sample grouping, microarray data pretreatment, statistical analysis and target gene predictions. To demonstrate the utility of RiceATM, we used the database to identify four miRNAs associated with the heading date and validated their expression trends in the cultivars with early or late heading date by real-time PCR. RiceATM is a useful tool for researchers seeking to characterize the role of certain miRNAs for a specific phenotype and discover potential biomarkers for breeding or functional studies.

**Database URL**: http://syslab3.nchu.edu.tw/rice/

## Introduction

Rice is an essential staple food worldwide. To manage problems stemming from global climate change and human population growth, breeders and scientists have been tasked with increasing rice yields. The yield components of rice have been identified and are known to be controlled by multiple genes (1–3), and these components have been utilized to improve rice production (4–6).

Similar to their mammalian homologues, plant microRNAs (miRNAs) can negatively regulate their target gene expression levels by perfect or imperfect binding to mRNAs in coding or untranslated regions. In general, miRNA can impact multiple genes ranging from a few to hundreds or even more targets, and it is an ideal regulator for multi-gene control mechanisms (7–9). These non-coding small RNAs with a functional sequence of 21–24 nucleotides (10, 11) are known to play important roles in plant developmental processes and stress-response regulation (12, 13). For example, a study identified 18 cold-responsive rice miRNAs, including miR167 and miR319, using a microarray approach on a single variety (14), and most of the differentially regulated genes were down-regulated in a cold-treated environment. Moreover, the rice yield-related gene OsSPL14, which is highly expressed in the reproductive stage and promotes panicle branching and higher grain yield, can be suppressed through excision by miR156 in Nipponbare (5). Almost all of the previous studies on rice miRNA have focused on one or a few cultivars. However, studies performing large-scale testing of rice cultivars and providing a parallel investigation of their miRNA expression profiles are not available.

Most of the published plant miRNA databases support mature and precursor miRNA sequences, miRNA gene coordinates, and miRNA target genes (15, 16). Certain plant miRNA databases reveal the association with phenotypes. For example, mirEX provides the miRNA profiles for seven different development stages (17), and PASmiR curates over 200 literature reports and indicates the effects of miRNA regulation under 35 abiotic stresses in 33 plant species (18, 19). However, the association between agronomic traits and miRNA expression profiles has not been well documented.

Rice breeding has been performed for over fifty years in Taiwan. Hundreds of cultivars, including japonica and indica rice, have been produced and provide the best genetic materials for breeding and research (http://tris.tari.gov.tw:8080/). In this study, a customized microarray was used to profile the expression patterns of 193 miRNAs in 187 rice cultivars with wide-ranging differences in agronomic traits, and rice agronomic traits and miRNA expression (RiceATM) platform (http://syslab3.nchu.edu.tw/rice/) was established to investigate the relationships between miRNA expression profiles and eight agronomic traits associated with rice yield. RiceATM allows users to obtain the significant miRNAs associated with a specific agronomic trait for use as biomarkers for breeding or functional studies.

## Materials and Methods

### Rice variety collection, cultivation and trait investigation

A total of 187 locally cultivated rice cultivars were collected from rice breeders located in the following four district agricultural research stations in Taiwan: Taichung, Kaohsiung, Taitung and Hualien. These cultivars were planted at the Agricultural Research Institute in Chia-Yi during the second crop season of 2009–10. The panicles were sampled 1–2 days before heading, immediately frozen in liquid nitrogen, and then stored at −80 °C until the total RNA could be isolated. The phenotypes after harvest were investigated according to standard procedures.

### Total and small RNA extraction

The panicle (5 g) was ground into powder in liquid nitrogen, and total RNA was isolated with extraction buffer as previously described. Total RNA was dissolved in distilled diethyl pyrocarbonate (DEPC) water and quantitated using a NanoDrop 1000 spectrophotometer V3.7 (Thermo Fisher Scientific; Wilmington, DE, USA) and then stored at −80ºC until miRNA isolation. Small RNAs were isolated using the PureLink miRNA Isolation Kit (K1570-01; Invitrogen/Thermo Fisher Scientific; Waltham, MA USA) according to the manufacturer’s instructions and quantitated by the NanoDrop system.

### miRNA microarray analysis

Because of the early initiation time of this study, we could only collect 193 miRNAs, and the identities were updated using the latest version of the miRBase database (version 21). The mature miRNA sequences and six control probes (four positive and two negative) were used to produce the customized rice miRNA microarray (Combimatrix Custom Array 4 × 2 K, CA, USA). Each miRNA probe was supplied in triplicate on the microarray, and each control probe contained five copies. For each rice cultivar, 2 μg of the purified small RNAs was employed to prepare the fluorochrome-labeled miRNAs (Cy5 Labeling Kit; Mirus Bio LLC, Madison, WI, USA) according to the manufacturer's instructions. Subsequently, the customized miRNA microarray was hybridized with the Cy5-labeled miRNAs in a 42ºC oven with slow microarray rotation for 4 h. After hybridization, the microarray was washed with a SSPE buffer series with 0.05% Tween-20 according to the manufacturer’s protocol (Combimatrix) and then subjected to image scanning and digitization using a GenePix 4000B scanner and GenePix 4.0 software (Molecular Devices) for further data analysis.

### Framework of RiceATM database

**Figure 1.**
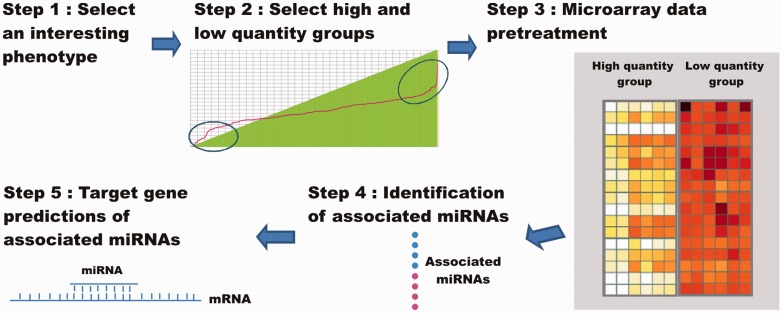
Architecture of the RiceATM platform. Step 1: Eight agronomic traits are represented in the RiceATM web server. The user can select an interesting trait and identify the associated miRNAs. Step 2: After selecting the agronomic trait, the user must fill in the ‘High cumulative percentage’ and “Low cumulative percentage” fields to identify the high- and low-quantity groups. The miRNA expression data on these two groups are selected for analysis. Step 3: In the microarray data pretreatment step, the user can select quantile normalization and data adjustment to normalize the microarray data. Step 4: To identify the miRNAs associated with the agronomic trait in the two groups of cultivars, RiceATM supports Student’s *t*-tests or ANOVAs. Step 5: Finally, the user can select the miRanda or psRNATarget algorithm to predict the target genes of the associated miRNAs.

We integrated the data for 8 agronomic traits and the expression of 193 mature miRNAs in 187 rice cultivars into a MySQL database on a CentOS system then used Java and the jQuery program to build the RiceATM platform, which can identify the associations between agronomic traits and miRNA expression profiles. The main steps for using the RiceATM platform are as follows (Figure 1):

*Step 1. Select the phenotype of interest*. This database provides the data on 8 reinvestigated agronomic traits in 187 locally cultivated rice cultivars for an association analysis. Users can select an interesting agronomic trait and determine the miRNAs that are associated with the trait.

*Step 2. Define the high-* *and low-quantity groups of rice.* To obtain a significant association between an agronomic trait and a miRNA expression profile, we provide users with the option to only obtain miRNA data from the highest and lowest quantity groups of cultivars for further analysis. The cut-off values for the high and low groups can be defined by the user or selected by the k-means algorithm. For example, if the user input 0.9 and 0.1 in the ‘High cumulative percentage’ and ‘Low cumulative percentage’ boxes, respectively, the database will automatically select the data for the two groups of sorted cultivars including percentages of 0.9–1 and 0–0.1, respectively.

*Step 3. Microarray data pretreatment.* In this step, the raw microarray data selected above can be subjected to quantile normalization (20), floor value assignment, and clipping values (min and max) according to the user’s selection.

*Step 4. Identification of miRNAs associated with an agronomic trait.* RiceATM utilizes ANOVAs and Student’s *t*-tests to identify the significantly differential expression of miRNAs between the two groups of cultivars. The false discovery rate (*Q*-value) of the test is estimated using the previously reported method (21).

*Step 5. Target gene prediction of identified miRNA.* The mature miRNA and mRNA sequences are downloaded from the miRBase database (version 21) (15) and the rice annotation project database (version 7.0) (22), respectively. The miRanda (ver. miRanda-Aug2010) (23) and psRNATarget web servers (24) are employed to predict the miRNA target genes with scores ≥ 160 and expectations ≥ 0, respectively. To visualize the relations between miRNAs and the target genes, the web server provides network user-interface using the selected miRNAs and their targets from the result of psRNATarget.

### miRNA reverse transcription and quantitative real-time PCR

For each reverse-transcription reaction, 2 μg of total RNA was reverse transcribed into cDNA using a miRNA-specific reverse transcription primer reverse transcriptase (Superscript III; Invitrogen, Carlsbad, CA) as previously described in (25). The miRNA expression level was detected using a real-time PCR reagent (FastStart SYBR Green Master, Roche) in a Rotor-Gene Q thermocycler (Corbett Research, Australia). The thermocycling program for the real-time PCR assay was as follows: 95 °C for 15 min (DNA polymerase activation), followed by 40 cycles of 94 °C for 15 s, 60 °C for 30 s and 72 °C for 30 s. Actin-11 was used as the internal control (26). The candidate miRNA expression that was normalized to the internal control expression was calculated as −ΔCT = −[CT_miRNA-Actin_]. The differences in the relative expression of the miRNA among cultivars were calculated using the 2^–ΔCT^ method. The PCR assays were performed in triplicate.

### Statistical analysis

All of the statistical tests in this study were performed using ANOVAs or Student’s *t*-tests with a two-tailed distribution in the software SAS 9.0 (version 9.1.3; SAS Institute, Cary, NC, USA). A *P* value < 0.05 was considered statistically significant. Where appropriate, the results are presented as the mean ± SD.

## Results

### Statistical data on the investigated agronomic traits

**Figure 2.**
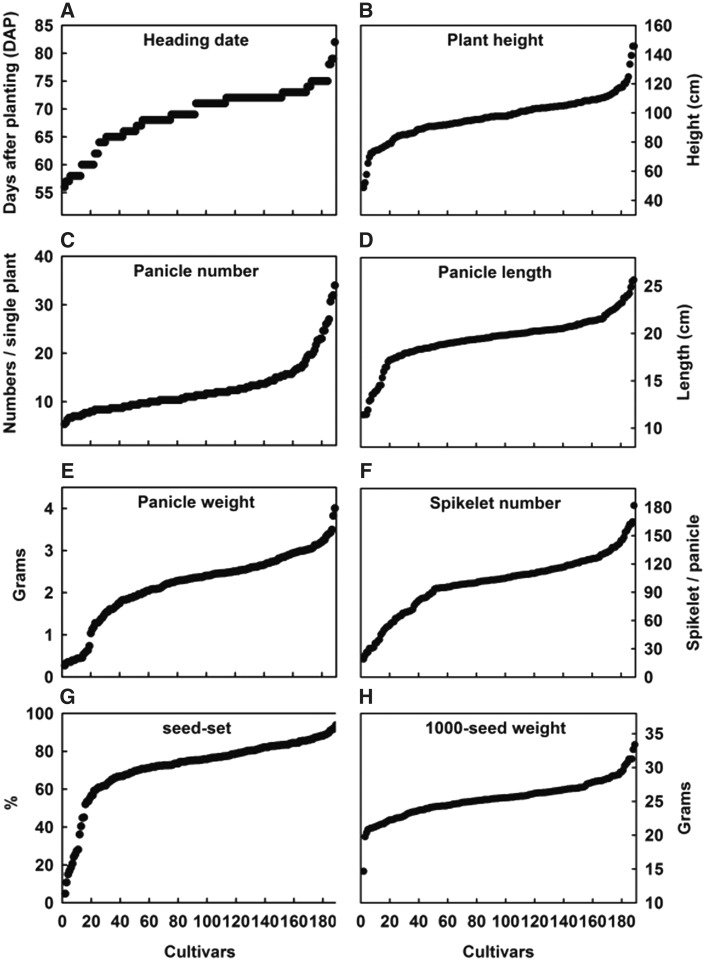
Line charts of 8 agronomic traits among 187 cultivars. **(A)** Heading data indicate the days after transplanting to paddy field. **(B)** Plant height indicates the average length from the bottom at the soil surface to the top of a single plant. **(C)** Panicle number indicates the average tiller number of a single plant. **(D)** Panicle length indicates the average length from the node of the panicle neck to the topmost single grain. **(E)** Panicle weight indicates the average weight of a single panicle. **(F)** Spikelet number indicates the average flower number of a single panicle. **(G)** Seed-set % indicates the percentage representing the ratio of developed seeds to total spikelets. **(H)** Thousand-seed weight indicates the total weight of 1000 rice grains.

Two types of rice cultivars were used in this study: japonica, *n* = 155, and indica, *n* = 32. These cultivars were cultivated in Chai-Yi County, and the mature panicles were sampled for further microarray or real-time PCR analysis. Three single plants of each cultivar were used to calculate the data for eight traits associated with rice yield, including the heading date, plant height, panicle number, panicle length, panicle weight, spikelet number, seed-set %, and 1000-seed weight. The collected data show the wide-ranging differences among the collected cultivars (Table 1). For example, the heading dates differ by one month between the earliest (56 days) and the latest (82 days) cultivar. Moreover, 2-fold differences occurred between the maximum and minimum values in the seven remaining traits. We sorted the measurements of each agronomic trait and plotted the line charts (Figure 2) and found that all of the line charts from the 8 agronomic traits among the 187 cultivars showed a similar ‘N’ shape, which indicates that a proportion of the cultivars fell within the middle of the distribution for certain phenotypes.

**Table 1.** Statistical data on the 8 investigated agronomic traits in 187 rice cultivars

	Average	Minimum	Maximum	Max./Min.
Heading date^1^	69.0	56.0	82.0	1.5
Plant height^2^	97.4	48.7	145.7	3.0
Panicle numbers^3^	12.5	5.3	34.0	6.4
Panicle length^4^	19.5	11.4	25.6	2.3
Panicle weight^5^	2.2	0.3	4.0	14.9
Spikelet numbers^6^	100.3	19.1	181.9	9.5
Seed-set%^7^	72.2	4.8	93.8	19.7
1000-seeds weight^8^	25.4	14.7	33.4	2.3

1 Units: days after transplanting;

2 centimeters;

3 panicles per single plant;

4 centimeter;

5 grams;

6 spikelet numbers per panicle;

7 ratio of matured seeds number to total spikelet numbers;

8 grams.

### miRNA profiles of rice cultivars by microarray

Microarray images were obtained using fixed scanning conditions (wavelength: 635 nm; PMT gain: 550; resolution: 5 μm pixel size) to avoid over-saturation and then digitized for subsequent analysis. The expression intensities of each miRNA were derived from the mean of triplicate probes. Thus, a total of 193 distinct measurements were obtained for each cultivar. After quantile normalization, the maximum and minimum values were 33428.4 and 73.3, respectively, which suggest significant differences occurred among the data in this database (see the download dataset). In addition, to measure the reproducibility of the miRNA microarrays, replicates of the miRNA probes were used to calculate the coefficient of variation (CV). The CV for the three replicates of each miRNA was 11.1 ± 4.8%, which was averaged over 193 miRNAs and 187 cultivars. The raw data were then employed to construct the RiceATM platform as described in the Methods section.

### Case studies

**Figure 3.**
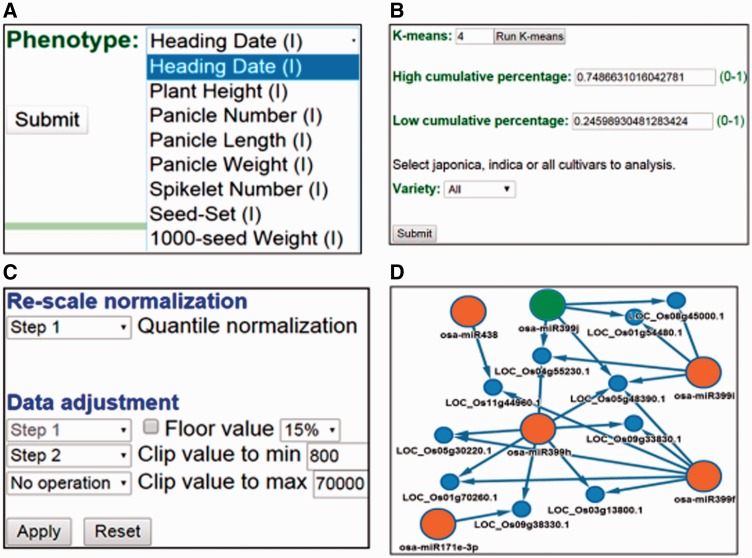
Example of browsing the RiceATM platform. **(A)** Eight agronomic traits affecting yield are represented in RiceATM, including the heading date, plant height, panicle number, panicle length, panicle weight, spikelet number, seed-set %, and 1000-seed weight. Here, we select ‘Heading Date’ as a demonstration. **(B)** RiceATM includes 187 rice cultivars: 155 japonica and 32 indica. The user can select total (japonica plus indica), japonica or indica cultivars to analyse by checking the ‘Variety’ box. In this example, we select the k-means clustering algorithm to select the high and low heading date groups for the total cultivars. **(C)** In the data pretreatment step, we use quantile normalization and then clip the minimum value at 800 to normalize the microarray data. **(D)** Differentially expressed miRNAs are evaluated by ANOVA and then subjected to target gene prediction by the psRNATarget algorithm. Thus, RiceATM shows the regulatory miRNA network. Large orange circles, miRNAs with high expression in the high-quantity group; large green circles, miRNAs with high expression in the low-quantity group; small blue circles, targeted mRNAs.

To demonstrate the RiceATM functions, we used the agronomic trait heading date as an example to test the pipeline (Figure 3A) (Supplementary Materials 1.2). After selecting the trait, we input four clusters in the k-means clustering algorithm to automatically identify the high- and low-quantity groups of cultivars (Figure 3B). The data from the selected microarrays were pretreated using the quintile method to normalize the data, and the minimum value was clipped at 800 (recommended by the chip manufacturer) (Figure 3C). Subsequently, an ANOVA was performed to identify significantly different miRNAs (*P* ≤ 0.05) between the two groups of cultivars. The RiceATM platform then output the miRNA signature associated with the heading date, including osa-miR172d-3p, osa-miR818c etc., sorted by *P* value (Table 2). To obtain the miRNA-regulatory network, we selected the psRNATarget algorithm (24) to predict the miRNA targets (Figure 3D). However, because miRanda predicted >100 target genes for each miRNA, its prediction results were not suitable for generating the network in this study. Users can input a miRNA ID or a RNA sequence to search the associated agronomic traits. When users input a RNA sequence, the web server will perform BLAST search to find the best match miRNAs, and then report the associated agronomic traits (Supplementary Materials 1.3).

**Table 2.** Gene list of miRNAs associated with the heading date phenotype as analysed by the RiceATM platform

Rank	Accession	Fold change (*H*_mean_/*L*_mean_)	*P* value	*Q* value
1	osa-miR172d-3p	1.201	1.534E-4	2.961E-2
2	osa-miR818c	1.304	7.608E-4	7.341E-2
3	osa-miR820c	1.136	6.240E-3	4.014E-1
4	osa-miR397a	1.200	6.451E-3	3.113E-1
5	osa-miR443	1.143	8.990E-3	3.470E-1
6	osa-miR438	1.203	9.988E-3	3.213E-1
7	osa-miR169c	0.757	1.054E-2	2.907E-1
8	osa-miR171h	1.252	1.060E-2	2.558E-1
9	osa-miR820b	1.161	1.169E-2	2.506E-1
10	osa-miR169i-5p.1	0.728	1.281E-2	2.472E-1
11	osa-miR821a	1.324	1.353E-2	2.374E-1
12	osa-miR172b	1.184	1.476E-2	2.374E-1
13	osa-miR395f	1.155	1.482E-2	2.201E-1
14	osa-miR440	1.201	1.520E-2	2.095E-1
15	osa-miR818b	1.087	1.679E-2	2.161E-1
16	osa-miR395y	1.051	1.838E-2	2.217E-1
17	osa-miR399h	1.199	2.016E-2	2.289E-1
18	osa-miR419	1.142	2.518E-2	2.700E-1
19	osa-miR395u	1.145	2.576E-2	2.617E-1
20	osa-miR415	1.159	2.623E-2	2.531E-1
21	osa-miR156e	0.807	2.715E-2	2.495E-1
22	osa-miR171c-5p	1.159	2.848E-2	2.498E-1
23	osa-miR812b	1.143	2.856E-2	2.397E-1
24	osa-miR399e	0.840	2.883E-2	2.318E-1
25	osa-miR418	1.172	3.206E-2	2.475E-1
26	osa-miR397b	1.118	3.291E-2	2.443E-1
27	osa-miR399f	1.198	3.426E-2	2.449E-1
28	osa-miR160b-5p	1.136	3.667E-2	2.528E-1
29	osa-miR171e-3p	1.120	3.935E-2	2.619E-1
30	osa-miR396a-5p	1.104	4.053E-2	2.608E-1
31	osa-miR169f.1	0.778	4.248E-2	2.645E-1
32	osa-miR156c-5p	0.834	4.613E-2	2.782E-1
33	osa-miR396b-5p	1.031	4.962E-2	2.902E-1

*H*_mean_, mean miRNA expression in the high-quantity group (early heading date); *L*_mean_, mean miRNA expression in the low-quantity group (late heading date).

In addition to the case of heading date mentioned above, two additional examples related to panicle development were provided to prove the usefulness of RiceATM. The miR397 has been reported to downregulate *OsLAC* expression, leading to increase in 1000-seed weight (27). Through the pipeline we built and the use of selection parameters (k-mean: 4; sample: all; default data pretreatment; and ANOVA), miR397 was identified as one of the significant miRNAs under the trait of 1000-seed weight (Supplementary Materials 2.1). Furthermore, the previous study revealed that miR156 is involved in panicle number regulation through targeting *OsSPL14* (5). By using the parameters we set (k-mean: 5; sample: all; default data pretreatment; and ANOVA), miR156 was identified as one of the significant miRNAs associated with the panicle number (Supplementary Materials 2.2).

### Validation of the selected miRNAs associated with heading date by quantitative real-time PCR

**Figure 4.**
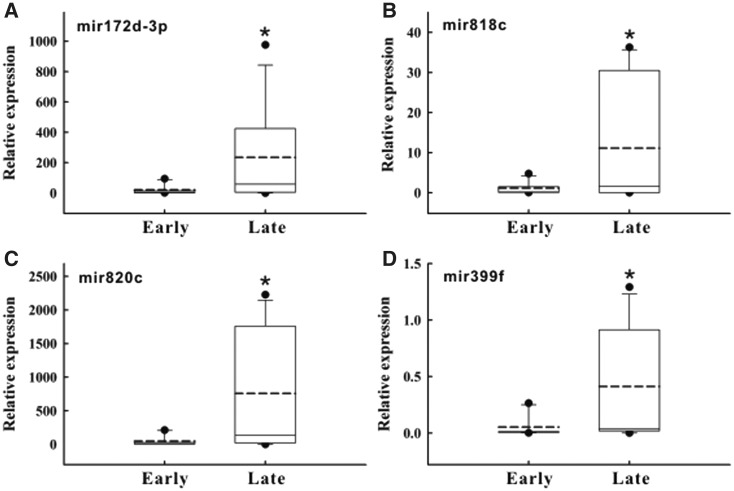
Expression trend of candidate miRNAs in the early and late heading date groups of rice cultivars. Four miRNA derived from RiceATM analysis and associated with heading date were subjected to a real-time PCR assay. Early, early heading date group, *n* = 4; Late, late heading date group, *n* = 4. Actin served as the internal control. **(A)** miR172d-3p; **(B)** miR818c; **(C)** miR820c and **(D)** miR399f. * *P* < 0.05, compared with the early group.

To confirm the accuracy of the RiceATM analysis and microarray data, a quantitative real-time PCR analysis was performed for the candidate miRNAs associated with heading date in eight cultivars, which included the 4 cultivars with the earliest heading date and the four cultivars with the latest heading date. The four miRNAs with positive fold changes identified above (Table 2; miR172d-3p, miR818c, miR820c and miR399f) were subjected to an expression-level analysis of the mature sequence. The results showed that the expression of these four miRNAs was significantly higher in the early heading date group than in the late heading date group (*P* < 0.05) (Figure 4), which is consistent with the microarray data.

## Discussion

miRNA has conserved functions in plant stress responses and developmental progression and is involved in regulating in multiple target genes in plants and animals (13, 23). Furthermore, miRNA signatures consisting of multiple miRNAs have been used to predict the clinical outcomes of lung cancer (28) and breast cancer (29) patients. In plant sciences, several studies have discussed the roles and influences of miRNAs on the organogenesis and traits of rice (9, 11, 13, 14); however, studies related to the relationships between phenotypes and miRNA expression profiling have not been published. It is generally believed that certain quantitative traits, such as heading date and panicle numbers, are controlled by multiple genes, and multi-targeting is one of the characteristics of miRNA. Thus, it is reasonable to assume that manipulating a small number of miRNAs would have the ability to modulate quantitative traits without requiring the control of multiple genes.

To our knowledge, previous reports have not shown the relationships between miRNA expression profiles and rice phenotypes in a large number of cultivars. Therefore, to build these relationships, we first investigated the eight agronomic traits associated with rice yield in 187 cultivars. Interestingly and unsurprisingly, all of the investigated phenotypes displayed a similar N-shaped pattern (Figure 2), implying that there extreme differences do not occur among approximately half of the cultivars. This phenomenon can also be observed in the agronomic trait data from the Taiwan Rice Information System (a database containing historical records of the phenotypes of cultivated rice in Taiwan). To effectively identify the differentially expressed miRNAs and eliminate the interference from distribution patterns of phenotypes, the RiceATM pipeline provides two options for isolating the high- and low-quantity groups: user-defined selection and k-means algorithm selection. The results of these selections are then subjected to significant difference analyses, such as *t*-tests and ANOVAs.

In general, plant miRNAs with similar mature sequences are grouped into the same family and may be involved in similar regulation pathways (10, 11). Therefore, only one primer set was designed for the quantitative real-time PCR analysis to verify the expression level of the miRNA if more than one member of a certain miRNA family was selected by the microarray screening. For instance, following the suggested operation procedures (Figure 3), the results indicated that 33 miRNAs were significantly associated with heading date (Table 2). The three top-ranked miRNAs with fold changes >1, namely miR172d, miR818c and miR820c, were selected for the real-time PCR validation. In addition, we found that the expression trends of the miR399 family members were extremely diverse; therefore, only miR399f, which presented a fold changes > 1, was selected for validation. Our data revealed that these four miRNAs were expressed more highly in the cultivars with late heading dates. This report is the first to identify the phenotype-related miRNAs from a large panel of cultivars with wide-ranging differences in agronomic traits. However, the action mechanisms and functional roles of these miRNAs in the regulation of heading date or rice yield require further investigation.

Among the heading date-related miRNAs, miR172 has been reported to be conserved and involved in flowering time and floral patterning by targeting AP2-like transcription factors across the monocotyledons and dicotyledons (30–32). In rice, miR172 was found to be highly expressed in the late vegetative stages and developing panicles. The overexpression of miR172b delayed the transition from spikelet meristem to floral meristem, thereby leading to defects in floral and seed development (33). Furthermore, a previous study indicated that the increased expression of miR172d resulted in the decreased expression of its target genes, SNB and OsIDS1, in phytochrome mutants as well as a delayed heading date in rice (34). In addition, several miR169 family members with fold changes < 1 were also selected by RiceATM screening. Although they were not used for validation in this study, several reports have shown that miR169 expression might be involved in changes to the root architecture (35) and the promotion of stress-induced flowering (36) by targeting the NF-YA transcription factor in *Arabidopsis thaliana*.

In summary, we utilized local rice cultivars with wide-ranging phenotypic differences and applied population genetics concepts to build the RiceATM platform, which has the potential to improve investigations into the correlations between miRNA expression levels and yield-related phenotypes in rice. For example, miR172, miR397 and miR156 that were previously discovered to associate with certain agronomic traits could also be identified in this database. RiceATM also has the potential to identify the role of certain miRNAs in specific phenotypes, and help researchers to focus their investigations, as and offer new tools to breeders researching breeding processes for trait improvement. However, the major limitation of RiceATM is the small number of miRNAs (only 193) used to construct the microarray, which may decrease the power of the results for gene screening on the whole-genome scale. Further updates and improvements, perhaps using next-generation sequencing approaches, are needed to increase the power of analysis.

## Availability

Database name: RiceATM (http://syslab3.nchu.edu.tw/rice/). All data deposited in the database are freely available to all users without any restrictions.
